# Correction: Khan et al. Examining the Association between Sports Participation and Mental Health of Adolescents. *Int. J. Environ. Res. Public Health* 2022, *19*, 17078

**DOI:** 10.3390/ijerph22040500

**Published:** 2025-03-26

**Authors:** Asaduzzaman Khan, Kazi R. Ahmed, Tarissa Hidajat, Tracy Kolbe-Alexander, Elizabeth J. Edwards

**Affiliations:** 1 School of Health and Rehabilitation Sciences, The University of Queensland, Brisbane 4072, Australia; 2 School of Education, The University of Queensland, Brisbane 4072, Australia; 3 School of Health and Medical Sciences, Centre for Health Research, University of Southern Queensland, Ipswich 4305, Australia; 4 UCT Research Centre for Health Through Physical Activity, Lifestyle and Sport (HPALS), Division of Research Unit for Exercise Science and Sports Medicine, University of Cape Town, Rondebosch 7700, South Africa; 5 Manna Institute, Centre for Health Research, University of Southern Queensland, Springfield Central 4300, Australia

Tracy Kolbe-Alexander was not included as an author in the original publication [1]. The corrected Author Contributions statement appears here.

**Author Contributions:** Conceptualization, A.K., K.R.A., T.K.-A. and E.J.E.; formal analysis, A.K. and K.R.A.; investigation, A.K., K.R.A., T.H., T.K.-A. and E.J.E.; resources, A.K.; data curation, A.K., K.R.A. and T.K.-A.; original draft preparation, A.K., K.R.A. and T.H.; review and editing, A.K., K.R.A. and E.J.E.; visualization, K.R.A.; supervision, A.K. and E.J.E. All authors have read and agreed to the published version of the manuscript.

In addition to affiliations 1 and 2, the updated affiliations should include:

3. School of Health and Medical Sciences, Centre for Health Research, University of Southern Queensland, Ipswich 4305, Australia
4. UCT Research Centre for Health Through Physical Activity, Lifestyle and Sport (HPALS), Division of Research Unit for Exercise Science and Sports Medicine, University of Cape Town, Rondebosch 7700, South Africa
5. Manna Institute, Centre for Health Research, University of Southern Queensland, Springfield Central 4300, Australia

The authors state that the scientific conclusions are unaffected. This correction was approved by the Academic Editor. The original publication has also been updated.
